# Age‐associated changes in the blood‐brain barrier: comparative studies in human and mouse

**DOI:** 10.1111/nan.12408

**Published:** 2017-05-29

**Authors:** E. F. Goodall, C. Wang, J. E. Simpson, D. J. Baker, D. R. Drew, P. R. Heath, M. J. Saffrey, I. A. Romero, S. B. Wharton

**Affiliations:** ^1^ Sheffield Institute for Translational Neuroscience The University of Sheffield Sheffield UK; ^2^ School of Life Science Health and Chemical Sciences Faculty of Science The Open University Milton Keynes UK

**Keywords:** Ageing, blood–brain barrier, human, mouse

## Abstract

**Aims:**

While vascular pathology is a common feature of a range of neurodegenerative diseases, we hypothesized that vascular changes occur in association with normal ageing. Therefore, we aimed to characterize age‐associated changes in the blood–brain barrier (BBB) in human and mouse cohorts.

**Methods:**

Immunohistochemistry and Evans blue assays were used to characterize BBB dysfunction (tight junction protein expression and serum plasma protein accumulation), vascular pathology (pericyte loss and vascular density) and glial pathology (astrocyte and microglial density) in ageing neurological control human prefrontal cortex (a total of 23 cases from 5 age groups representing the spectrum of young adult to old age: 20–30 years, 31–45 years, 46–60 years, 61–75 years and 75+) and C57BL/6 mice (3 months, 12 months, 18 months and 24 months, *n* = 5/6 per group).

**Results:**

Quantification of the tight junction protein ZO‐1 within the cortex and cerebellum of the mouse cohort showed a significant trend to both increased number (cortex *P* < 0.001, cerebellum *P* < 0.001) and length (cortex *P* < 0.001, cerebellum *P* < 0.001) of junctional breaks associated with increasing age. GFAP expression significantly correlated with ageing in the mice (*P* = 0.037). In the human cohort, assessment of human protein accumulation (albumin, fibrinogen and human IgG) demonstrated cells morphologically resembling clasmatodendritic astrocytes, indicative of BBB dysfunction. Semiquantitative assessment of astrogliosis in the cortex expression revealed an association with age (*P* = 0.003), while no age‐associated changes in microglial pathology, microvascular density or pericyte coverage were detected.

**Conclusions:**

This study demonstrates BBB dysfunction in normal brain ageing, both in human and mouse cohorts.

## Introduction

The mechanisms underlying central nervous system (CNS) dysfunction and cognitive deterioration during ageing are complex. It is likely, however, that blood–brain barrier (BBB) dysfunction has a contributory role in the ageing process. The BBB is formed by a continuous layer of cerebral endothelial cells (CECs) held together by tight junctions, which limit paracellular and intramembranous diffusion. This restricts penetration of molecules into the CNS and provides high electrical resistance 1. The ‘sealing’ feature of the tight junctions is due to the unique expression of specific transmembrane proteins, including occludin and junction adhesion molecules (JAMs) 2, 3. Scaffolding proteins, such as zonula occludens‐1 (ZO‐1), link the tight junction complex to the cytoskeleton 4, 5. As a result the molecular traffic of endogenous substances, necessary to maintain CNS homeostasis, occurs mainly by transcellular means. Transport of small molecules, such as glucose and amino acids, requires the expression of specific transporters, whereas larger molecules, such as peptides, cross the BBB by transcytosis. Other transporters specific to CECs, such as P‐glycoprotein (also known as multidrug resistance protein 1 or ATP‐binding cassette subfamily B member 1), are specialized for efflux of a wide range of substrates and contribute to removal of harmful substances from within the CNS 6. The phenotype of CECs is under inductive influence of perivascular pericytes and the end‐feet of astrocytes. Together, these cells form a functional cellular complex termed the ‘neurovascular unit’ (NVU), which provides a coordinated response to maintain CNS homeostasis and function 6.

Leakage of the BBB, associated with changes in the expression of tight junction proteins, has been found in various acute and chronic neurological disorders 7, 8, 9, 10, 11. However, to date, studies investigating the effect of normal, physiological ageing on the integrity of the BBB are conflicting, with some studies concluding the BBB remains intact 12, 13, 14, 15, 16, while others show evidence of increased permeability 17, 18, 19. A systematic review suggests that BBB breakdown may be common in normal human ageing as evidenced by increased cerebrospinal fluid/plasma albumin ratios and brain imaging studies 20. Indeed, our studies in an ageing population‐representative neuropathology cohort show that BBB leakage is a common feature of the cerebral cortex 21 and white matter in the ageing human brain 22, but these studies were confined to the elderly population (>65 years).

To further investigate the effects of ageing on NVU integrity through adult life, we determined BBB permeability in cohorts of mice and an ageing human autopsy cohort, in addition to microvascular density, glial pathology and vascular pathology in the human cohort. Previous studies have shown leakage of serum proteins, such as albumin, fibrinogen and IgG, to be robust markers of impaired BBB function 23, 24, therefore we used albumin‐binding dyes and immunohistochemistry to visualize leakage in mice and human aged tissues respectively. Our findings provide evidence of an age‐associated decline in BBB function which may contribute to the vulnerability of the aged CNS to neurodegenerative disease.

## Material and methods

### Study cohort: Mouse

C57BL/6 mice were purchased from Charles River at 3 months (young adult), 12 months (adult), 18 months (middle age) and 24 months (old). All mice were sacrificed and perfused with phosphate‐buffered saline (PBS). The brain and spinal cord were dissected, then either formalin fixed and paraffin embedded (FFPE) (*n* = 5 per group), snap frozen in liquid nitrogen or used for Evans blue assay (*n* = 6 per group), as described below. All animal experiments were conducted following ethical review processes in accordance with the Animals (Scientific Procedures) Act 1986 of the UK government and the ARRIVE guidelines 25.

### Study cohort: Human

*Post mortem* human brain tissue was obtained from the Edinburgh Medical Research Council sudden death brain bank, who selected the cases and granted approval for the use of tissue in this study (Edinburgh Brain Bank REC reference 11/ES/0022). FFPE tissue was obtained from the prefrontal association cortex (Brodmann areas 8/9) of cases without a history of neurological disease selected from five age groups to cover the spectrum of young adult to old age, namely 20–30 years, 31–45 years, 46–60 years, 61–75 years and 75+. Five cases were obtained per group, except for the 75+ group, where only three non‐neurological cases were available. Case details are provided in Table 1. Haematoxylin and eosin (Cellpath, UK) stained sections of human cortex were examined from each case by a neuropathologist and immunohistochemistry was carried out with antibodies to phospho‐tau (AT8) and β‐amyloid to document age‐associated pathological changes in the tissue.

**Table 1:** Human brain bank cases demographics

Age group	Age	Gender	*Post mortem* interval (h)	Cause of death	Neuropathology
20–30	20	F	71	Suspension by ligature	None
	24	F	47	Suspension by ligature	Small vessel disease/perivascular spaces and arteriosclerosis
	25	M	53	Suspension by ligature	None
	29	M	44	Suspension by ligature	Focal tau, no neuritic plaques
	30	M	71	Unascertained	None
31–45	32	F	63	Cardiomyopathy/Marfan's syndrome	None
	36	M	41	IHD	None
	39	F	43	Suspension by ligature	None
	42	M	61	IHD	None
	44	M	47	Drug overdose	None
46–60	46	M	52	IHD	None
	48	M	72	CAD	None
	50	M	45	IHD/CAD	None
	52	M	91	Road traffic collision	None
	57	M	66	IHD/CAD	None
61–75	63	F	35	IHD/CAD	Mild amyloid tangles and plaques
	70	F	79	Ruptured aneurysm	Large vessel arteriosclerosis and ischaemic white matter pathology. Mild tau threads and tangles. Mild vascular amyloid, tangles and plaques
	71	F	41	IHD	Mild and focal vascular tau. Mild amyloid tangles and plaques
	74	M	46	Pulmonary thromboembolism	None
	74	M	66	IHD/CAD	White matter pallor, mild tau tangles, plaques and treads. Mild vascular amyloid, tangles and plaques
75+	75	M	78	IHD/CAD	Mild tau tangles and treads
	76	M	90	CAD/Myocardial infarction	Mild tau tangles and treads
	79	F	45	IHD/CAD	Venous collagenosis and small vessel disease

IHD, ischaemic heart disease; CAD, coronary artery disease.

### Evans blue assay

Evans blue (Sigma, UK), 2% in PBS at 80 mg/kg, was administered intraperitoneally 24 h before mice from each age group were anaesthetized with sodium pentobarbital and sacrificed (*n* = 6 per group). Intracardiac perfusion was then performed through the left ventricle with 100 ml of 0.9% ice‐cold saline using a peristaltic pump (speed 7.5 ml/min) to remove intravascular Evans blue. Blood was collected from the right ventricle by cardiac puncture. Brain and spinal cord were dissected, weighed and homogenized in PBS. Then, 2.5 ml of trichloroacetic acid (Sigma, UK) was added and samples were incubated on ice for 30 min before centrifugation at 1000 **g** at 4°C for 30 min. Absorbance of supernatants and plasma (dilution factor 100x) were measured at 610 nm. The results were normalized to wet weight of tissue and plasma concentration of Evans blue and expressed as (ml of plasma)/(g of tissue).

### Immunohistochemistry

Immunohistochemistry was performed using a standard avidin–biotin complex–horse radish peroxidase (ABC‐HRP) method, and visualized with diaminobenzidine (Vector Laboratories, UK), except for albumin immunohistochemistry which was performed using a standard ABC–alkaline phosphatase method, and visualized with alkaline phosphatase red substrate (Vector Laboratories, UK). Both isotype controls and no primary antibody controls were included in every run. A summary of all the primary antibodies and their conditions of use is shown in Table 2. All immunohistological evaluation of both the human and mouse ageing cohorts was performed blind to any clinical information.

**Table 2:** Antibody sources and experimental conditions

Antibody	Tissue	Isotype	Dilution (time, temp)	Antigen retrieval	Supplier
Tau (AT8)	Human FFPE	Mouse IgG	1:400 (o/n, 4°C)	MW 10 min, TSC pH 6.5	Endogen, UK
β‐amyloid	Human FFPE	Mouse IgG	1:100 (o/n, 4°C)	MW 10 min, TSC pH 6.5^a^	DakoCytomation, UK
Albumin	Human FFPE	Rabbit IgG	1:15 000 (1 h, RT)	MW 10 min, TSC pH 6.5	DakoCytomation, UK
Fibrinogen	Human FFPE	Rabbit IgG	1:3000 (1 h, RT)	MW 10 min, TSC pH 6.5	DakoCytomation, UK
IgG	Human FFPE	Rabbit IgG	1:32 000 (1 h, RT)	PC, EDTA pH 8	DakoCytomation, UK
CD31	Human FFPE	Rabbit IgG	1:100 (1 h, RT)	MW 10 min, TSC pH 6.5	AbCam, UK
GFAP	Human FFPE	Rabbit IgG	1:1000 (1 h, RT)	MW 10 min, TSC pH 6.5	DakoCytomation, UK
IBA1	Human FFPE	Mouse IgG	1:200 (1 h, RT)	MW 10 min, TSC pH 6.5	Millipore, UK
ZO‐1	Mouse frozen	Rabbit IgG	1:100 (o/n, 4°C)	N/A	Invitrogen, UK
CD31	Mouse frozen	Rat IgG	1:10 (o/n, 4°C)	N/A	BD Bioscience, BD Pharmingen

FFPE, formalin‐fixed paraffin embedded; o/n, overnight; MW, microwave; TSC, trisodium citrate; RT, room temperature; PC, pressure cooker; EDTA, ethylenediaminetetraacetic acid; N/A, not applicable.

a Antigen retrieval carried out following pretreatment with formic acid for 5 h.

#### Plasma proteins

BBB leakage in human tissue was determined using immunohistochemistry to plasma proteins: albumin (67 kDa), fibrinogen (340 kDa) and immunoglobulin G (IgG, 150 kDa). Staining patterns were scored semiquantitatively in the area of most intense immunoreactivity (×20 objective) and independently scored by a second observer to assess interobserver variability. Assessment was based on proportions of positive neurons, parenchymal/perivascular staining and white matter staining, using schemes modified from our previous studies in cortex and white matter (illustrated in 21, 22). In the cortex, neuronal immunoreactivity was assessed as none (1), isolated (2), frequent, representing up to approximately 50% of neurons (3) or very frequent, representing more than 50% (4). Parenchymal/perivascular immunoreactivity was scored as none (1), patchy (2) or confluent (3). In the white matter, serum protein immunoreactivity was graded as follows: some blood vessels displaying evidence of perivascular albumin deposition (1), weak glial reactivity in addition to the perivascular deposition (2), many positive glial cells within the parenchyma (3) and intense, confluent perivascular and parenchymal albumin reactivity with many positive glia (4).

#### Tight junction proteins

Immunohistochemistry to zonula occludens‐1 (ZO‐1) was carried out to identify breaks in tight junctions, using the primary–secondary method, and visualized with Alexa Fluor^®^ 594‐conjugated secondary antibody. In the mouse tissue, Z‐stacks of 30 vessels were imaged from both the cerebral cortex and cerebellum of each mouse, using Zeiss confocal microscopy and analysed by Image J software. In brief, the outline of individual vessels was traced from deconvoluted Z‐stack images and plot profiles displayed as pixel distance against grey value. Tight junction integrity was assessed by measuring break length per 100 μm of microvessel, number of breaks per 100 μm of microvessel and average break length. The break was registered when the grey value was lower than the threshold determined by using mean of background ± 2 standard deviations in Image J software.

#### NVU cells

Assessment of specific immunoreactivity was performed by capturing bright‐field microscopic images in three adjacent 350‐μm‐wide cortical ribbons, consisting of contiguous fields to cover the total cortical thickness, using a ×20 objective (Nikon Eclipse Ni‐U microscope, Nikon, UK) and analysed using the Analysis ^D software (Olympus Biosystems, Watford, UK). To further assess microvascular changes in the human cohort, microvascular density was assessed using immunohistochemistry to CD31. Percentage CD31 area immunoreactivity and intervessel pixel distance were quantitated using Analysis D software. Percentage area pericyte coverage of capillaries was assessed in a subset of old (*n* = 6, aged over 70) and young human cases (*n* = 5, aged 30 and below) using double staining: periodic acid–Schiff (PAS) to stain vessels and immunohistochemistry to PDGFRβ to specifically label pericytes. Astroglial and microglial responses in perivascular regions and neuropil were examined with immunohistochemistry to GFAP and Iba1 respectively.

### Statistical analysis

Statistical analyses were performed using IBM SPSS Statistic v20 and GraphPad Prism v6. Spearman's rank correlation was used to assess the relationship between histoscores and age. Interobserver variability was assessed using weighted kappa. Trend analyses for age groups were performed using the Jonckheere–Terpstra test (JT) unless otherwise stated. Evans blue and ZO‐1 data were not normally distributed and did not show equality of variances between groups, therefore analyses were performed using nonparametric methods. The relationship of these markers to age was determined using Kruskal–Wallis test (KW), and the presence of trends analysed using the JT test. Pericyte coverage data were analysed using unpaired *T* test, following confirmation of normal distribution of data via Shapiro–Wilk test.

## Results

### Loss of ZO‐1 expression is associated with ageing in the mouse brain

**Figure 1:**
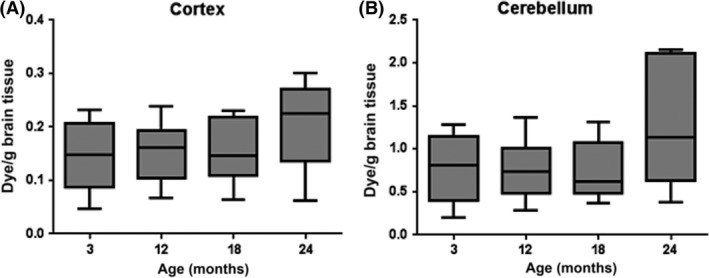
BBB permeability in mice. Quantification of Evans blue did not detect significant age‐associated BBB changes in the (**A**) cortex or (**B**) cerebellum of an ageing mouse cohort.

**Figure 2:**
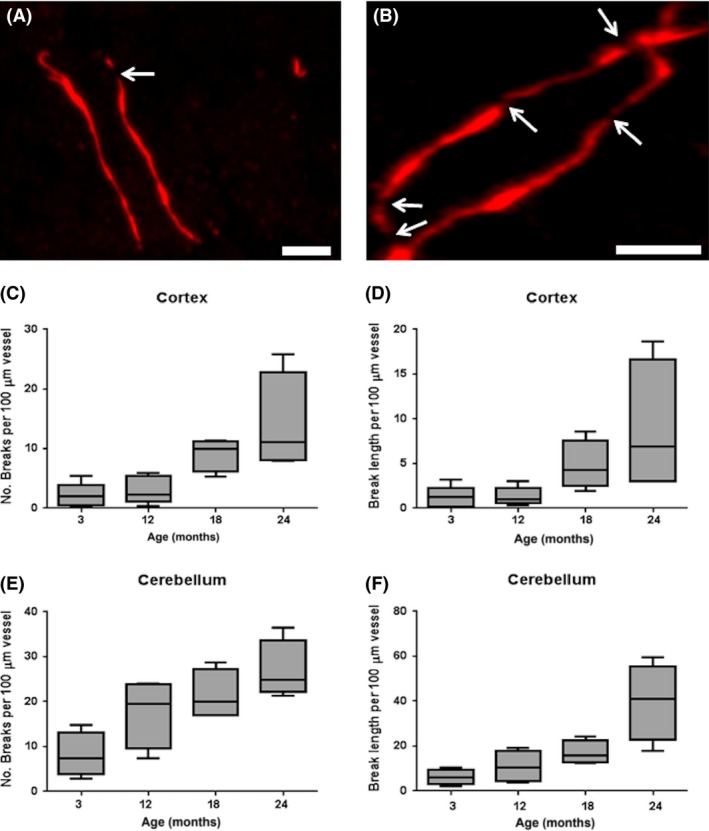
Age‐associated loss of ZO‐1 in mice. (**A**) ZO‐1 immunolabelling of tight junctions in a young mouse (3 months). (**B**) Breaks in endothelial ZO‐1 expression were quantitated in the ageing mouse cohort, as indicated by the white arrows (24 month case). A significant trend to increased (**C**) number (*P* < 0.001) and (**D**) length (*P* < 0.001) of tight junction protein breaks associated with age in the cortex. A significant age‐associated increase in (**E**) the number (*P* < 0.001) and (**F**) length (*P* < 0.001) of tight junction protein breaks were also detected in the cerebellum. Scale bar represents 5 μm in **A** and **B**.

To quantify BBB permeability across our aged cohort of mice we used albumin‐binding Evans blue dye assays. Under normal conditions, with an intact BBB, the albumin–dye complex cannot enter the CNS; when the BBB is disrupted, neighbouring tissues stain blue. Significantly more Evans blue was detected in the cerebellum compared to the cortex across all ages (*P* < 0.001) (Table S1), suggesting regional differences in BBB permeability. However, within the cortex there was no significant difference between age groups (KW *P* = 0.49) and no trend to increased Evans blue extravasation associated with age (JT *P* = 0.237) (Figure 1 **A**). Similarly, within the cerebellum there was no significant difference between age groups (KW test *P* = 0.45) and no trend to increased Evans blue extravasation associated with age (JT *P* = 0.237) (Figure 1 **B**). ZO‐1 staining patterns revealed linear outlines of tight junctions along microvessels (Figure 2 **A**) with focal areas of loss representing breaks in the BBB (Figure 2 **B**). Within the cortex and cerebellum quantification of the number of breaks in the BBB showed a significant difference between groups (KW: cortex *P* = 0.012, cerebellum *P* = 0.025), with a significant trend to increased number of breaks associated with age (JT: cortex *P* < 0.001, cerebellum *P* < 0.001) (Figure 2 **C,E**). Within the cortex and cerebellum quantification of BBB break length showed a significant difference between groups (KW: cortex *P* = 0.011, cerebellum *P* = 0.019), with a significant trend to increased break length associated with age (JT: cortex *P* < 0.001, cerebellum *P* < 0.001) (Figure 2 **D**,**F**).

### Human case characterization across the ageing cohort

Haematoxylin and eosin‐stained sections demonstrated some white matter pallor in five cases, all over 50 years of age. Diffuse and compact amyloid plaques, with occasional amyloid in vessels were seen in four cases (age 63–74 years) and vascular amyloid was seen in two of these four cases. Mild tau pathology was observed in four cases, all aged over 70 years of age. These tau and amyloid pathologies are very commonly seen in normal ageing 26, 27. One young case (29 years) showed a single focus of tau pathology without neuritic plaques (Figure S1) and a second case in this youngest group showed some widening of perivascular spaces and arteriolosclerosis, suggestive of mild small vessel disease.

### Increased extrusion of serum proteins is associated with ageing in the human brain

Quantification of tight junction breaks was not performed in the human tissue. Although this method has been successful in our hands in another human autopsy cohort 21, 22, we obtained only diffuse endothelial staining in this human cohort, without the clear pattern of intercellular junctions, suggesting artefactual degradation of these membrane‐associated proteins. Therefore, further analysis was not attempted using tight junction markers in the human tissue.

**Figure 3:**
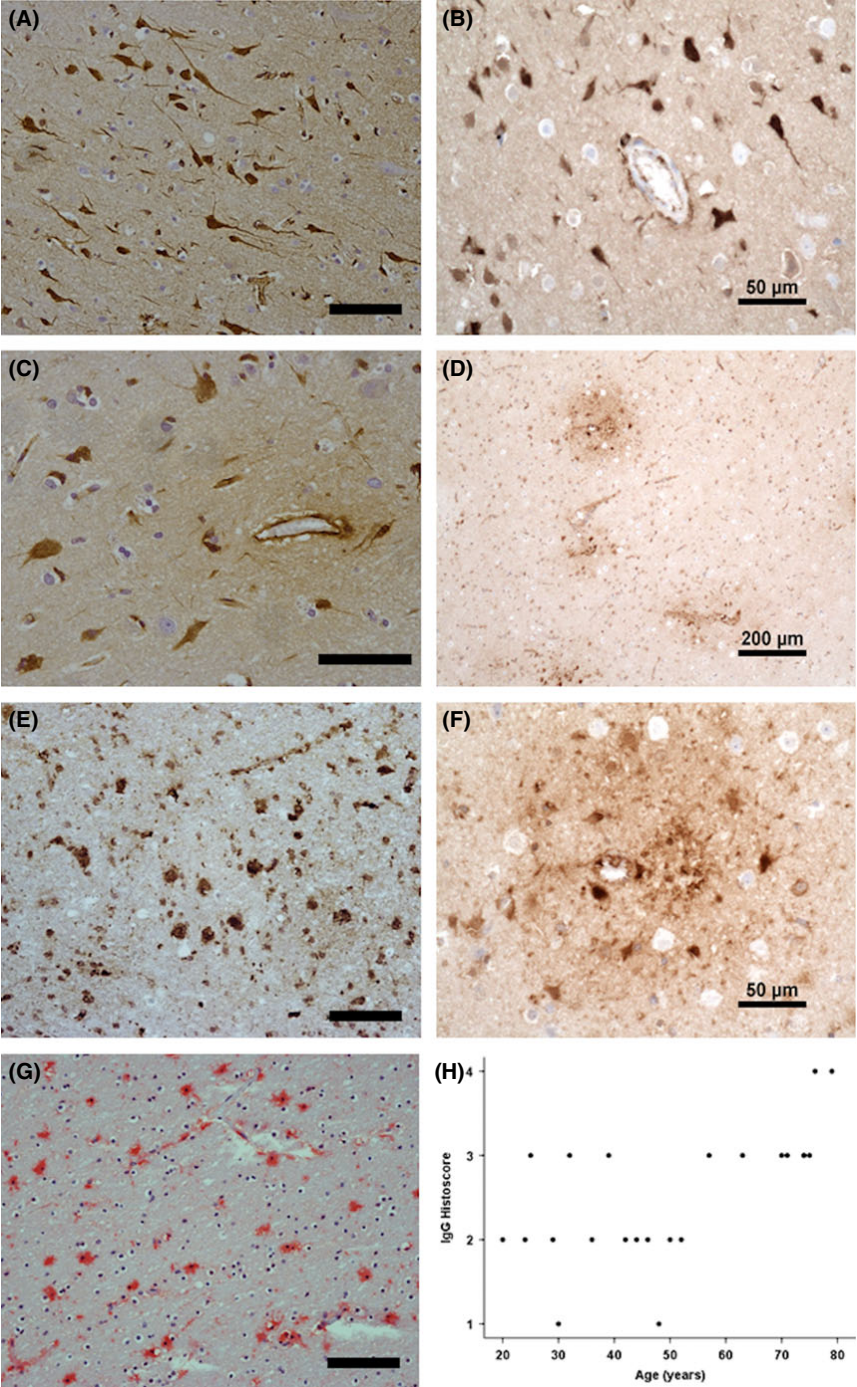
Serum protein accumulation in the ageing brain. (**A**) Fibrinogen and (**B**) IgG immunopositive neurons. (**C**) Fibrinogen and (**D**) IgG perivascular immunostaining. (**E**) Fibrinogen, (**F**) IgG and (**G**) albumin clasmatodendritic astrocytes were observed in the ageing human cohort. (**H**) IgG immunoreactivity in the white matter positively associated with age (*P* = 0.002). Scale bar represents 100 μm in **A**,**E**,**G**; 50 μm in **B**,**C**,**F** and 200 μm in **D**.

Immunohistochemistry to three serum proteins (albumin, fibrinogen and IgG) showed a variety of staining patterns with variation between cases, as shown in Figure 3. Both fibrinogen and IgG immunopositive neurons were detected in the cortex (Figure 3 **A,B**). In white matter, perivascular and diffuse staining patterns were seen (Figure 3 **C,D**), with some cases containing large, round immunopositive cells morphologically recognizable as clasmatodendritic astrocytes (Figure 3 **E,G**). Staining of the subpial region (layer 1) was widespread across the cohort with little variation (therefore, layer 1 was not included in quantification). Immunostaining of IgG in the white matter showed a strong positive association with ageing (JT test, *P* = 0.002) (Figure 3 **H**), while neuronal and vascular IgG scores did not correlate with ageing. The score of the first observer was used for analysis, with the score of the second observer used to check interobserver variation in scoring. Neuronal IgG staining showed moderate agreement between scorers (κ = 0.491), vascular and white matter scoring showed low agreement between scorers (κ = 0.169 and κ = 0.152 respectively), suggesting that these patterns are harder to assess, with consequently less interobserver reliability. The mean white matter IgG score of the two observers also showed an increase with age (JT *P* = 0.004). Initial analysis of fibrinogen did not reveal a significant relationship with age. However, we noticed a trend towards fibrinogen increase with age, except in the youngest group, where fibrinogen scores were also high. This group (Table 1) had mostly died by suspension ligature, raising the question of whether *perimortem* raised venous pressure might have contributed to higher leakage at this age. In addition, we observed unusual pathology in two of the cases within this group, specifically, a focal region of tau immunoreactivity (S1) and white matter pathology. We therefore repeated the analysis, excluding the youngest age group. Fibrinogen now also showed an age relationship in both the grey (*P* = 0.011) and white matter (*P* = 0.044). Albumin, with the lowest molecular weight, showed more extensive distribution in the human tissue and no correlation with increasing age. We examined the relationship of fibrinogen and IgG leakage to *post mortem* delay in our cohort and did not find a significant correlation (data not shown).

### Age‐associated changes in glial pathology

**Figure 4:**
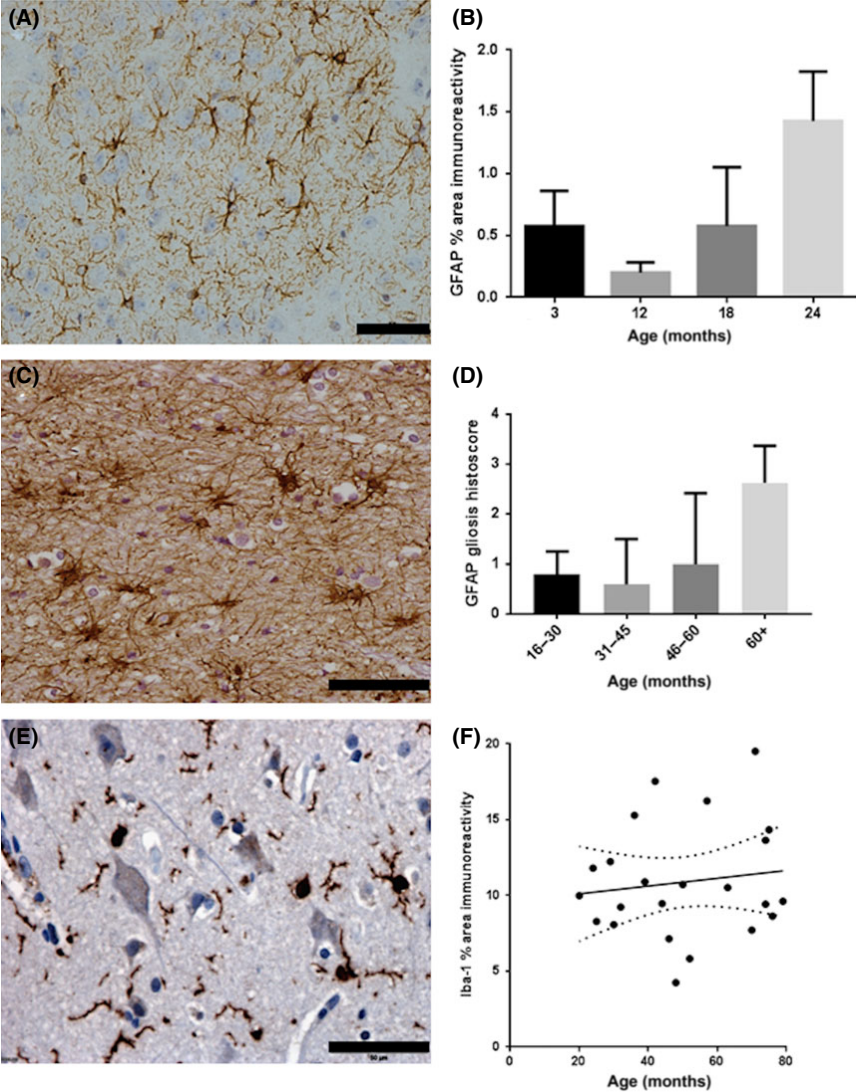
Age‐associated changes in glial pathology. (**A**) GFAP immunolabelling of astrocytes in the mouse brain. (**B**) GFAP area immunoreactivity correlates with age in all but the youngest age group of the mouse cohort (*P* = 0.016). (**C**) GFAP immunolabelling of astrocytes in the human brain. (**D**) In the ageing human cohort, semiquantitative assessment of gliosis in the cortex expression demonstrates an association with age (*P* = 0.003). (**E**) Iba‐1^+^ microglia in the human brain. (**F**) In contrast to GFAP, no age‐associated changes in Iba‐1 expression were detected. Scale bar represents 50 μm in **A**,**C**,**E**.

GFAP^+^ astrocytes with a classic stellate morphology were detected in the mouse cohort (Figure 4 **A**). Levels of GFAP expression, assessed as percentage area immunoreactivity, showed a significant increase in the mice with ageing (KW *P* = 0.037) (Figure 4 **B**). A JT trend test on these data was not significant. However, the plots show that GFAP is higher at 3 months, then drops before increasing steadily with age. If the 3‐month group is excluded, then the JT test showed a significant increase with age (*P* = 0.016). GFAP expression was also examined in the human ageing cohort (Figure 4 **C**). No increase in GFAP percentage reactivity with age was observed in the human tissue samples; however, a variety of staining patterns were observed. We therefore used semiquantitative analysis to assess for gliosis alone and observed a significant increase with age in humans (JT *P* = 0.003) (Figure 4 **D**). Notably, gliosis was a prominent feature in the over 60 years old cases, but we did not detect increasing perivascular gliosis with ageing. Iba‐1^+^ microglia were also examined in the human cohort (Figure 4 **E**). The percentage area immunoreactivity of Iba‐1 did not increase with ageing and perivascular microgliosis was not detected (Figure 4 **F**).

### No age‐associated changes in microvascular density or pericyte coverage

**Figure 5:**
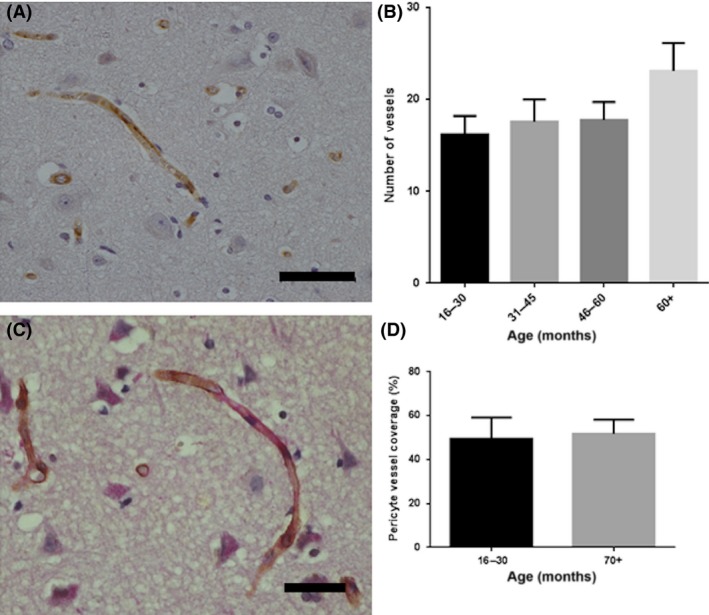
Age‐associated changes in vascular pathology. (**A**) CD31 immunolabelling of cerebral vessels in the human cortex. (**B**) No significant age‐associated changes in microvascular density were detected. (**C**) PCGFRβ^+^ pericytes (brown) are intimately associated with vessels (PAS, red). (**D**) There was no significant difference in the pericyte coverage of vessels in the youngest *vs*. the oldest group. Scale bar represents 50 μm.

No changes in the pattern of immunostaining of the endothelial marker CD31 (either area immunoreactivity for CD31 or numbers of vessels) or intervascular pixel distance were observed in the human tissue (Figure 5 **A,B**). Pericyte coverage of vessels was compared in young (mean age = 26.7 years) *vs*. old (mean age = 74.8 years) subcohorts. There was no significant difference in percentage pericyte coverage between the two groups (*P* = 0.97, 10df, mean difference 0.34%, 95% CI: −23.4 to 22.72) (Figure 5 **C,D**).

## Discussion

In the current study, we present evidence of microvascular changes associated with normal ageing in both mouse and human cohorts. Evans blue extravasation in the mouse cohort was significantly higher in the cerebellum than the cortex across all ages. The finding of regional differences supports morphometric evidence for age‐associated regional variation, suggesting certain brain areas are more susceptible to BBB breakdown than others 28, 29. These variations may contribute to the inconsistent outcomes of BBB permeability studies carried out in ageing to date 20. Changes in the expression of the tight junction protein ZO‐1 are closely associated with the degree of BBB damage 30. Using more rigorous quantitative analysis of the tight junction protein ZO‐1, we demonstrate a highly significant increase in BBB breaks throughout the life of our mouse cohort. As the loss of ZO‐1 gradually accumulated over time and occurred at earlier time points, this suggests a physical disruption to tight junctions and the resulting increased paracellular flux is a contributor to the consequent leakage. ZO‐1 protein levels are dynamic and respond to physiological and pathological conditions, such as hypoxia, oxidative stress and inflammation, which are features of normal ageing and could potentially be driving BBB leakage 31, 32, 33.

We also observed a gradual age‐associated increase in astroglial activation in the mouse cohort. These changes preceded events that we may consider as pathological, such as BBB leakage, which occurred at old age in the mice. GFAP levels were also higher in 3‐month‐old mice, supporting a role for the protein during development 34. These findings highlight the importance of looking longitudinally to understand brain ageing and find intervention targets.

Although immunohistochemical detection of ZO‐1 has been successful in other human autopsy cohorts 21, 22, 35, in the current study only diffuse endothelial staining was observed with no clean signal along endothelial cell boundaries, suggesting artefactual degradation. Therefore, immunohistochemistry to serum proteins was employed to assess BBB leakage in the ageing human brain. In contrast to the mouse cohort, cortical changes were less clear in our human cohort and there was considerable population variation. Increased fibrinogen extravasation positively associated with age in all but the youngest. In this youngest age group, 4/5 died by suspension, suggesting that raised venous pressure *perimortem* might have driven serum protein accumulation. This group also showed other minor pathological features, gliosis and some focal tau in one case, the significance of this is uncertain as these cases had no neurological disease but may suggest subtle brain abnormalities in this group.

Although the majority of ageing research is focussed on cortical pathology, white matter vascular changes have also been detected by imaging 36, 37 and shown to contribute to cognitive decline in the ageing population, as recently reviewed 38. In the current study, we demonstrate increased vascular leakage in the ageing white matter, particularly IgG, accompanied by the presence of clasmatodendritic astrocytes. These serum protein‐positive astrocytes have a swollen, rounded form with loss or fragmentation of processes and are considered to reflect BBB leakage in white matter 39, 40. A consequence of clasmatodendrosis is a decrease in astrocyte end‐feet coverage of blood vessels which could compromise the neurovascular unit and was recently shown to associate with cognitive decline following stroke 41.

Breakdown of the BBB leads to accumulation of circulatory molecules (such as inflammatory molecules and serum proteins) within the brain. The observed uptake of serum proteins in neuronal cell bodies and glia within our cohort support this, and such molecules may stimulate a neuroinflammatory response 42. We demonstrate an increase in gliosis associated with ageing, in keeping with the changes in GFAP expression observed in the mouse cohort. We did not observe a change in the number or morphology of perivascular microglia, however, future studies are required to assess levels of microglial activation, for example, MHC II expression.

There have been several vascular density studies in ageing (reviewed in detail in Ref. 43) and overall results have been mixed with regional differences and variable results in neurodegeneration. In the current study, microvascular density was not affected by normal ageing in the brain regions studied. Previous studies have found evidence of pericyte loss with ageing associated with BBB breakdown 44. Although we did observe loss of pericyte coverage in the human cohort, there was no correlation with age. However, we examined relatively few cases and, given the wide confidence intervals for percentage pericyte coverage, a larger study is required.

A limitation of any study using human autopsy material is *post mortem* sampling, with results difficult to interpret as they can be affected by agonal events, population variation, coexisting pathology and *post mortem* uptake of serum proteins. The current study selected non‐neurological cases, but causes of sudden death varied between age groups, which may be most relevant in the youngest group, and subclinical age‐related pathology may vary. Thus, pathological examination revealed some instances of white matter pallor and tau pathology as expected in an ageing brain. *Post mortem* leakage of serum proteins may also occur from delay in autopsy 45, although we did not find a relationship with *post mortem* delay in our cohort. Because of the difficulties in examining BBB changes in human autopsy tissue, we carried out parallel studies in mice. The comparability of findings provides further evidence that this is a process generally relevant to brain ageing, and that the mouse is a good model to study age‐related BBB and NVU changes.

Overall this study provides evidence for BBB dysfunction in the normal brain ageing process and indicates importance for both white matter and cortex. Collectively, our data suggests that disruption to the BBB may initiate processes contributing to declining function. Changes in some of the cellular and molecular components of the BBB and NVU appear to develop progressively with ageing. More accurate measures of BBB integrity could therefore be a biomarker that predicts poorer brain ageing and therefore increased risk of cognitive decline. Defining processes related to the BBB and NVU may yield predictive and intervention targets that may be of value in mid‐life as well as at older ages as part of efforts to improve healthy brain ageing.

## Author contribution

SBW and IAR conceived and designed the experiments. EF, CW, JES, DJB and DRW performed the experiments. SBW, IAR, EFG, CW and JES analysed the data. EFG, CW, JES, PRH, MJS, IAR and SBW wrote the paper.

## Disclosure

All authors have seen and approved the manuscript. There are no conflicts of interest specific to this manuscript.

## Supporting information


**Figure S1.** Case characterization in the ageing cohort. Focal region of tau pathology without neuritic plaques, observed in one human *post mortem* case aged 29 years. Scale bar represents 200 μm.

**Table S1.** Quantitation of Evans blue extravasation, number of ZO‐1 breaks and length of BBB breaks (median [interquartile range]) in the cortex and cerebellum of an ageing mouse cohort.
