# Supplementary figures and images for: Age‐associated changes in the blood‐brain barrier: comparative studies in human and mouse
Source: Neuropathol Appl Neurobiol. 2017 May 29;44(3):328–40. doi: 10.1111/nan.12408 (PMC5900918; doi:10.1111/nan.12408)

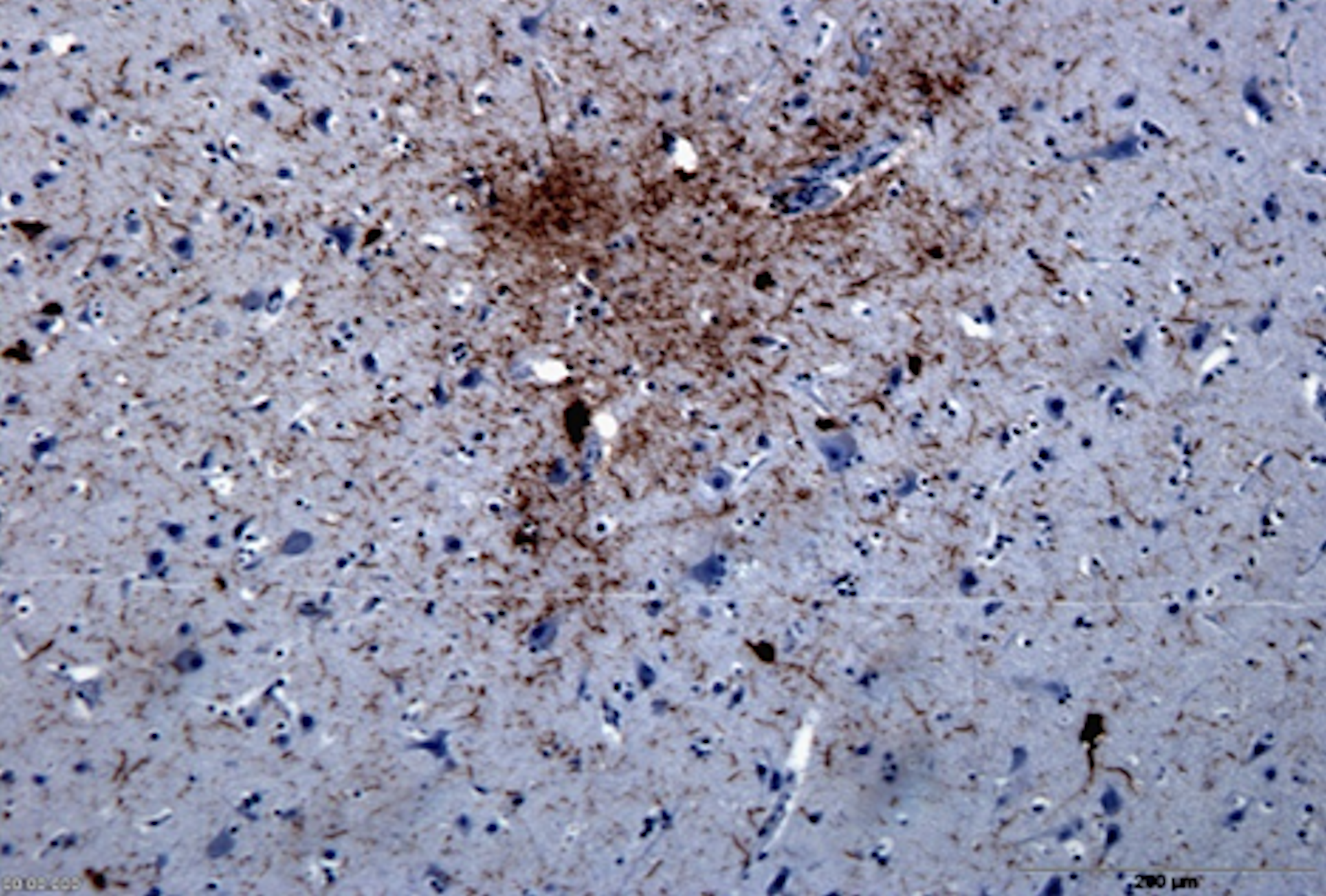

Supplement: Supplementary file 1 — Figure S1. Case characterization in the ageing cohort. Focal region of tau pathology without neuritic plaques, observed in one human post mortem case aged 29 years. Scale bar represents 200 μm. [file NAN-44-328-s001.tif]
