# Supplementary material for: Age‐associated changes in the blood‐brain barrier: comparative studies in human and mouse
Source: Neuropathol Appl Neurobiol. 2017 May 29;44(3):328–40. doi: 10.1111/nan.12408 (PMC5900918; doi:10.1111/nan.12408)
Supplement: Supplementary file 2 — Table S1. Quantitation of Evans blue extravasation, number of ZO‐1 breaks and length of BBB breaks (median [interquartile range]) in the cortex and cerebellum of an ageing mouse cohort. [file NAN-44-328-s002.docx]

**Supplementary Table 1.** Quantitation of Evans Blue extravasation, number of ZO-1 breaks and length of BBB breaks (median [inter-quartile range]) in the cortex and cerebellum of an ageing mouse cohort.

| **Age Group (Months)** | **3** | **12** | **18** | **24** |
| --- | --- | --- | --- | --- |
| Cortex dye/g tissue | 0.13 (0.06-0.19) | 0.16 (0.09-0.18) | 0.14 (0.08-0.20) | 0.22 (0.10-0.25) |
| Cerebellum dye/g tissue | 0.81 (0.28-1.09) | 0.73 (0.38-0.86) | 0.62 (0.43-0.90) | 1.14 (0.51-1.96) |
| Cortex break length/0.1mm | 0.71 (0.14-1.30) | 1.17 (0.48-2.61) | 4.27 (2.48-7.56) | 6.85 (3.03-16.67) |
| Cortex break number/0.1mm | 1.32 (0.41-2.26) | 3.44 (0.80-5.71) | 9.93 (6.23-11.26) | 11.05 (8.03-22.77) |
| Cerebellum break length/0.1mm | 6.16(2.95-9.31) | 10.34 (4.42-17.94) | 15.93 (12.87-22.65) | 40.94 (22.80-55.60) |
| Cerebellum break number/0.1mm | 7.40 (3.82-13.13) | 19.55 (9.55-23.80) | 20.07 (17.03-27.20) | 24.92 (22.21-33.66) |
